# A Novel Tax-Responsive Reporter T-Cell Line to Analyze Infection of HTLV-1

**DOI:** 10.3390/pathogens13111015

**Published:** 2024-11-19

**Authors:** Stefanie Heym, Pauline Krebs, Kristin Ott, Norbert Donhauser, Laura M. Kemeter, Florian Simon, Sebastian Millen, Andrea K. Thoma-Kress

**Affiliations:** Institute of Clinical and Molecular Virology, Friedrich-Alexander-Universität Erlangen-Nürnberg (FAU), 91054 Erlangen, Germany; stefanie.heym@uk-erlangen.de (S.H.); kristin.ott@fau.de (K.O.); norbert.donhauser@uk-erlangen.de (N.D.); laura.kemeter@uk-erlangen.de (L.M.K.); florian.simon@uk-erlangen.de (F.S.);

**Keywords:** HTLV-1, Tax, reporter T-cell line, *U3R*, Tax-responsive element, HTLV-1 promoter, infection, HTLV-1 cell-to-cell transmission, SMPU

## Abstract

Human T-cell leukemia virus type 1 (HTLV-1) infects CD4^+^ T-cells through close cell–cell contacts. The viral Tax-1 (Tax) protein regulates transcription by transactivating the HTLV-1 *U3R* promoter in the 5′ long terminal repeat of the integrated provirus. Here, we generated a clonal Tax-responsive T-cell line to track HTLV-1 infection at the single-cell level using flow cytometry, bypassing intracellular viral protein staining. Jurkat T-cells stably transduced with the SMPU vector carrying green fluorescent protein (GFP) under control of 18 × 21 bp Tax-responsive element repeats of the *U3R* were evaluated. Among 40 clones analyzed for Tax responsiveness, the top two were characterized. Upon overexpression of Tax, over 40% of the cells showed GFP positivity, and approximately 90% of the Tax-positive cells were GFP-positive, indicating efficient reporter activity. However, with CREB-deficient Tax mutant M47, both total GFP-positive cell counts and those within the Tax-positive group significantly decreased. Co-culture with chronically HTLV-1-infected MT-2 or C91-PL cells led to an average of 0.9% or 2.4% GFP-positive cells, respectively, confirming the suitability to monitor HTLV-1 transmission and that HTLV-1 infection is very low. Thus, the novel Tax-responsive reporter T-cell line is a suitable tool to monitor infection of HTLV-1 on the single-cell level.

## 1. Introduction

Human T-cell leukemia virus type 1 (HTLV-1) is a highly oncogenic delta-retrovirus and the cause of severe diseases like adult T-cell leukemia/lymphoma (ATLL) or inflammatory conditions like HTLV-1-associated myelopathy/tropical spastic paraparesis (HAM/TSP), which may develop in about 3–5% of infected carriers after lifelong viral persistence [1]. The main target cells of HTLV-1 in vivo are CD4^+^ T lymphocytes. Transmission of HTLV-1 to T-cells requires cell–cell contacts, while cell-free transmission is inefficient [2]. Cell-to-cell transmission occurs at close cell–cell contacts via polarized budding of viral particles towards target cells at virological synapses and via extracellular viral assemblies, so-called viral biofilms [3,4]. Transmission towards far distantly located cells is accomplished by the formation of cellular protrusions and tunneling nanotubes, which are induced by the accessory viral protein p8 [5,6,7]. The HTLV-1 receptor is composed of heparan sulfate proteoglycans, neuropilin-1 (Nrp-1), and the ubiquitously expressed glucose transporter 1 (Glut-1) [8,9,10]. The HTLV-1 receptor components Glut-1 and Nrp-1 are also upregulated on activated T-cells [10,11], thus making them more prone to HTLV-1 cell-to-cell transmission.

Upon de novo infection of an HTLV-1 target cell, the viral RNA is reversely transcribed followed by integration into the host cell’s DNA. The integrated proviral genome is flanked by *long terminal repeats* (LTRs) comprising *unique 3* (U3), *redundant* (R), and *unique 5* (U5) regions harboring enhancer/promotor elements to control transcription of *HTLV-1*. Upon activation of the 5′ *LTR*, viral genes encoding Gag, Pol, and Env as well as regulatory and accessory genes are transcribed [12]. Amongst regulatory proteins, the viral transactivator Tax is a crucial regulator of viral sense transcription by transactivating the *U3R* promotor in the 5′ *LTR*. Within the *U3* region, enhancer elements consisting of three 21 bp direct repeats are located, namely, the *Tax-response elements* (TREs) [13,14,15,16,17]. Each repeat contains three conserved domains (A, B, C), which account for 13 nucleotides out of 21. Domain B forms the *viral cAMP responsive element* (vCRE), which is particularly important for Tax-mediated transactivation. Initiation of viral transcription takes place at the *vCRE* site, which is mediated by the binding of a protein complex consisting of the cAMP response element binding protein (CREB), Tax, and the CREB-binding protein (CBP) or p300 [17,18,19,20,21]. Subsequently, viral gene expression is boosted, resulting in the production of Tax and the aforementioned viral proteins. Thus, the production of Tax is a measure of de novo infection upon cell-to-cell transmission.

Important insights into HTLV-1 transmission were gained by imaging techniques, revealing not only the different pathways of viral transmission but also the localization of viral and cellular proteins during this process [2,3,4,7,22,23,24,25,26,27]. To quantitatively measure HTLV-1 cell-to-cell transmission, several groups reported the use of *LTR*-based reporter vectors ranging from *LTR*-driven luciferase or GFP reporter cells [28], *U3R*-driven luciferase reporter cells [23], viral *CRE*-driven luciferase plasmids [29], or *LTR*-driven NanoLuc reporter systems [30]. Reporter vectors had either been transfected or delivered via retroviral vectors into target cells prior to co-culture experiments with HTLV-1-infected donor cells. While being useful for obtaining insights into the molecular details of HTLV-1 transmission, previously used reporter cell lines face several limitations. (1) Although being sensitive, luciferase-based reporter cell lines cannot discriminate single cells, thus making it impossible to analyze and characterize infected cells in more detail. (2) Systems using the *LTR*/*U3R* viral promoter, although being closer to reality, might overlook rare events of virus transmission, which may be visible when using an artificial but stronger viral promoter. (3) It is not clear from every study whether they used clonal or polyclonal reporter cell lines. Repeated use of the latter may limit the reproducibility of data obtained due to differences in the growth properties of different clones depending on the integration site of the transgene.

To boost the readout of Tax-induced signals on the *HTLV-1* promoter, Zhang and colleagues developed an *enhanced green fluorescent protein* (eGFP) reporter vector called SMPU driven by 18 copies of the 21 bp *TRE* repeats of the HTLV-1 *U3R* promotor region. The transduction of different cell lines using self-inactivating lentiviral vectors (SIN) led to high titers of viruses that integrated into the chromosomes of different cell types [31]. This 18 × 21 bp-eGFP reporter SMPU seemed to be a promising starting point for the generation of a novel clonal reporter cell line to monitor HTLV-1 transmission on the single-cell level. Here, we generated and characterized a clonal Tax-responsive reporter cell line to detect HTLV-1 infection on the single-cell level by flow cytometry whilst avoiding intracellular staining of viral proteins. Jurkat T-cells were transduced with SMPU, and single clones thereof were analyzed for their responsiveness towards Tax by measuring eGFP expression and intracellular Tax protein in parallel. Finally, co-cultures of pre-stained reporter clones with chronically HTLV-1-infected MT-2 or C91-PL cells showed that the clones are suitable tools to monitor HTLV-1 cell-to-cell transmission, albeit confirming that HTLV-1 infection is very low.

## 2. Materials and Methods

### 2.1. Cell Culture

Jurkat T-cells and Jurkat–SMPU cell clones derived thereof were cultured in RPMI 1640 medium (Thermo Fisher Scientific, Carlsbad, CA, USA) supplemented with 45% Panserin 401 (PAN Biotech GmbH, Aidenbach, Germany), and 293T cells were maintained in DMEM (Thermo Fisher Scientific). Chronically HTLV-1-infected C91-PL and MT-2 cells were cultured in RPMI 1640 medium. All cell culture media were supplemented with 10% fetal calf serum (FCS; Anprotec, Bruckberg, Germany), 1× GlutaMAX, and penicillin/streptomycin (all Thermo Fisher Scientific). Cell lines were tested for mycoplasma contamination. C91-PL cells have been described earlier and were obtained from Maria-Luisa Calabrò (University of Padova, Padua, Italy) [32]. Jurkat T-cells, 293T cells, and MT-2 cells have been used before and were kindly provided by Ralph Grassmann (deceased, FAU, Erlangen, Germany) [5].

### 2.2. Stimulation of Cells

To mimic T-cell activation, Jurkat–SMPU cell clones were treated with Phorbol 12-myristate 13-acetate (PMA; 20 nM; Sigma-Aldrich, St. Louis, MO, USA) and ionomycin (Iono; 1 µM; Merck Millipore, Darmstadt, Germany) or phytohaemagglutinin P (PHA-P; 2 µg/mL; Sigma-Aldrich) and interleukin-2 (IL-2; 25 U/mL; Roche Diagnostics, Basel, Switzerland) for one to seven days.

### 2.3. Plasmids and Cloning

The following plasmids were used: the lentiviral vector SMPU-18 × 21-EGFP encoding eGFP under control of 18 repeats of the 21 bp Tax-responsive element (TRE) was kindly provided by Dr. Chou-Zen Giam [31]; psPAX2 (Addgene plasmid #12260) and pMD2.G encoding vesicular stomatitis virus glyocoprotein G (VSV-G; Addgene plasmid #12259) were provided by Didier Trono (http://n2t.net/addgene:12259 (accessed on 13 November 2024); RRID:Addgene_12259; http://n2t.net/addgene:12260 (accessed on 13 November 2024); RRID:Addgene_12260; both accessed on 13.11.2024); pSG5-Tax encoding Tax under control of a simian virus 40 (SV 40) promotor [33]; pSG5 (Agilent Technologies, Santa Clara, CA, USA); and pSG5-M47 was cloned by substituting the Tax wildtype cDNA fragment of psG5-Tax with the cDNA fragment encoding Tax M47 from pc-M47 [34] using EcoRI and BamHI restriction enzymes and standard cloning techniques. The primer pairs EcoRI-Tax-fwd (5′-ATTA*GAATTC*GCCACCATGGCCCACTTCCCA-3′) and BamHI-Tax-rev (5′-ATAT*GGATCC*TCAGACTTCTGTTTCGCGGA-3′) were used to amplify M47 cDNA. All plasmids were checked for integrity by Sanger DNA sequencing and/or diagnostic digestion.

### 2.4. Transient Transfection

For transient transfection of 293T cells, 0.5 × 10^6^ 293T cells were seeded in 6-well plates one day prior to transfection and incubated overnight at 37 °C. GeneJuice Transfection Reagent (Merck Millipore, Darmstadt, Germany) was applied according to the manufacturer’s protocol, and a total amount of 2 µg of DNA was used. Cells were analyzed at 48 h after transfection. For transient transfection of Jurkat T-cells or Jurkat–SMPU reporter T-cell clones, 10 × 10^6^ cells were transfected with a total amount of 100 μg plasmid DNA using the Gene Pulser Xcell^®^ Electroporation System (BioRad, München, Germany) as previously described [35]. Briefly, electroporation cuvettes with an electrode distance of 4 mm (Peqlab, Erlangen, Germany) were used and an exponential pulse (290 V, 1500 μF, resistance infinite) was applied. Cells were analyzed at 48 h after transfection.

### 2.5. Generation of Jurkat Cell Clones Containing a Stably Integrated Tax-Responsive 18 × 21-eGFP Reporter Cassette (Jurkat–SMPU)

To derive a stable Jurkat reporter T-cell line monitoring HTLV-1 infection, the SMPU-18 × 21-eGFP vector was employed [31]. Jurkat T-cells were transduced as described previously [27]. Briefly, for lentiviral production, 5 × 10^6^ 293T cells were seeded in 10 cm dishes 24 h prior to transfection, which was conferred using GeneJuice transfection reagent and a total amount of 15 µg DNA: 6 μg SMPU-18 × 21-EGFP vector, 6 μg HIV-1 gag-pol expression vector psPAX2, and 3 μg VSV-G expression vector pMD2.G. At 72 h post-transfection, lentivirus-containing supernatant was placed in Amicon^®^ Ultra^®^-15 Centrifugal Filter (Ultracel-100 K; Merck Millipore) and concentrated at 4000× *g* for 15 min. Then, 1 × 10^6^ Jurkat T-cells were spin-infected with 1.5 ml concentrated virus (150 g, 2 h, 32 °C) and adjusted to 2 × 10^5^ Jurkat cells/mL. Approximately ten days after transduction, stable Jurkat cells were sorted for single cells into 96 well U-bottom plates using MoFlo™ XDP High-Speed Sorter (Beckman Coulter, Indianapolis, IN, USA). Resulting Jurkat–SMPU single-cell clones were expanded, and each clone was tested for functionality by electroporation of a Tax expression plasmid (pSG5-Tax) or respective controls (see Section 2.4) and subsequent detection of GFP signals by flow cytometry.

### 2.6. Immunofluorescence

Epifluorescence images of Jurkat–SMPU cells were obtained 48 h after transfection with the Nikon Eclipse TS2-FL Inverted Microscope and the TCapture software (Version 5.1.1.0, Tucsen, Fuzhou, China).

### 2.7. Flow Cytometry

For detection of eGFP expression, 293T or Jurkat cells were washed in PBS (1200 rpm, 5 min, 20 °C) prior to fixation in 2% para-formaldehyde (PFA) for 20 min at 20 °C. For flow cytometry, cells were resuspended in PBS and measured at the BD LSR II flow cytometer (BD Biosciences, Heidelberg, Germany). For intracellular co-staining of Tax, transfected Jurkat cells or Jurkat–SMPU reporter cell clones were washed twice in PBS (1200 rpm, 5 min, 20 °C) before fixation in 2% PFA. After 20 min at 20 °C, cells were washed first in PBS, 2 mM EDTA, 1% FCS followed by a washing step in wash buffer (PBS, 2 mM EDTA, 1% FCS, 0.3% saponin). Cells were stained using mouse anti-Tax antibodies (1A3; Abcam, Cambridge, UK; 1:2000) or IgG2 isotype control antibodies (Abcam) diluted in staining buffer (PBS, 2 mM EDTA, 1% FCS, 0.5% saponin) for 30 min at 20 °C. After two washing steps in wash buffer, cells were incubated in staining buffer containing secondary antibodies anti-mouse Alexa Fluor 647 (Life Technologies, Carlsbad, CA, USA; 1:2000) for 30 min at 20 °C. After two final wash steps in wash buffer, cells were resuspended in PBS and analyzed at the Attune NxT flow cytometer (Thermo Fisher Scientific). All data generated by flow cytometry were analyzed using FlowJo^TM^ software (Version 10.8.1, BD Biosciences).

### 2.8. Western Blot Analysis

Proteins were extracted at 48 h post-transfection. Cells were washed twice in PBS (5 min, 1200 rpm, 20 °C) and lysed in 150 mM NaCl, 10 mM Tris (pH 7.0), 10 mM EDTA, 1% Triton X-100, 2 mM DTT, 20 μg/mL leupeptin, 20 μg/mL aprotinin, and 1 mM PMSF. Subsequently, freeze–thaw lysis was performed in two cycles. The samples were therefore frozen in liquid nitrogen and thawed at 30 °C and 1400 rpm on a shaker. Afterwards, the samples were sonicated by using the Branson Sonifier 450 (Emerson Electric Co., St. Louis, MO, USA; duty cycle: 80%, output control: 8; 3 × 20 s, 4 °C). Cellular debris was removed by centrifugation (14,000 rpm, 4 °C, 15 min) and protein concentrations were determined in protein-containing supernatants by the Bradford protein assay using the Roti^®^-Quant reaction agent (Carl Roth GmbH + Co. KG, Karlsruhe, Germany). Equal amounts of protein lysates per experiment (30–50 µg) were diluted 1:5 in sodium dodecyl sulfate (SDS) loading buffer (10 mM Tris (pH 6.8), 10% glycerol (*w*/*v*), 2% SDS (*w*/*v*), 0.1% bromphenolblue (*v*/*v*), and 5% β-mercaptoethanol (*v*/*v*)) and boiled at 95 °C for 5 min. For SDS polyacrylamide gel electrophoresis (SDS-PAGE), samples were loaded on 12% polyacrylamide gels alongside 5 µL of PageRuler^TM^ Prestained Protein Ladder (Thermo Fisher Scientific), and gels were run at 130 V for approximately 1 h in 1:10 diluted SDS running buffer (25 mM Tris, 250 mM glycine, 0.1% SDS (*w*/*v*)) using the X-CellSureLock^®^ Mini-Cell System (Life Technologies). After separation, proteins were transferred onto nitrocellulose membranes using the Mini-Protean^®^ Tetra System (BioRad) in a 1:10 dilution of 25 mM Tris and 192 mM glycine for 75 min at 250 mA. Afterwards, membranes were incubated in blocking solution containing a 1:10 dilution of TBS (150 mM NaCl, 2 mM Tris/HCl, pH 7.6), supplemented with 5% FCS and 0.1% Tween^®^ 20 (*v*/*v*) for 1 h. Primary antibodies mouse anti-Tax (derived from the hybridoma cell line 168B17-46-34, provided by B. Langton through the AIDS Research and Reference Reagent Program, Division of AIDS, NIAID, NIH) [36] or mouse anti-GAPDH (H2521; 1:1000; Santa Cruz Biotechnology, Dallas, TX, USA) were incubated in blocking solution at 4 °C overnight. Membranes were washed three times in washing solution (1× TBS, 0.1% Tween [*v*/*v*]) and incubated with horseradish peroxidase (HRP)-conjugated secondary antibodies anti-mouse IgG HRP (1:2000; GE Healthcare, Chicago, IL, USA) in blocking solution for 30 min at 20 °C. After the final washing steps, peroxidase activity was detected using enhanced chemiluminescence and the Advanced Fluorescence Imager (ChemoStar; Intas Science Imaging GmbH, Göttingen, Germany).

### 2.9. Co-Culture Assays to Monitor Infection with HTLV-1

Jurkat–SMPU reporter T-cell cones 21 and 29 and the mixed clone of 21, 29, 31, and 34 were stimulated with 25 U/mL IL-2 and 2 µg/mL PHA-P. After 48 h, stimulated acceptor cells were stained with CellTrace^TM^ Violet according to the manufacturer’s protocol (CTV; Thermo Fisher Scientific) and used as acceptor cells in co-culture experiments (2.5 × 10^5^ cells/mL). Prior to co-culture, HTLV-1-infected donor cell lines MT-2 and C91-PL were irradiated at 77 Gray (Gy) using Gammacell 2000 (Molsgaard Dental A/S, Copenhagen Science Park Symbion, Copenhagen, Denmark). In total, 5 × 10^5^ cells/mL were co-cultured at a ratio of 1:1 in 48-well plates. On days 0 and 3 of co-culture, cells were additionally stimulated with 25 U/mL IL-2. As negative controls, either stimulated or unstimulated Jurkat T-cells were used instead of HTLV-1-infected donor cells. Flow cytometry analysis of eGFP reporter activity in CTV+ cells was performed on days 0, 3, and 6 of co-culture. Briefly, co-cultures were spun down, washed in PBS (5 min, 1200 rpm, 20 °C), and fixed in 2% PFA for 60 min at 20 °C. After the resuspension of cells in PBS, cells were analyzed at the AttuneNxt flow cytometer. Data were analyzed using FlowJo^TM^ software.

### 2.10. Statistics

Data were analyzed for normal distribution using the Shapiro–Wilk Test followed by an unpaired Student’s *t*-test (two groups) or one-way ANOVA and Tukey’s test (more than two groups) using GraphPad Prism (GraphPad Software, Version 9.5.1, Boston, MA, USA). *p* values < 0.05 (*) were considered significant (** *p* ≤ 0.01, *** *p* ≤ 0.001, and **** *p* ≤ 0.0001).

## 3. Results

### 3.1. Generation of the Tax-Responsive Jurkat–SMPU Reporter Cell Line

**Figure 1:**
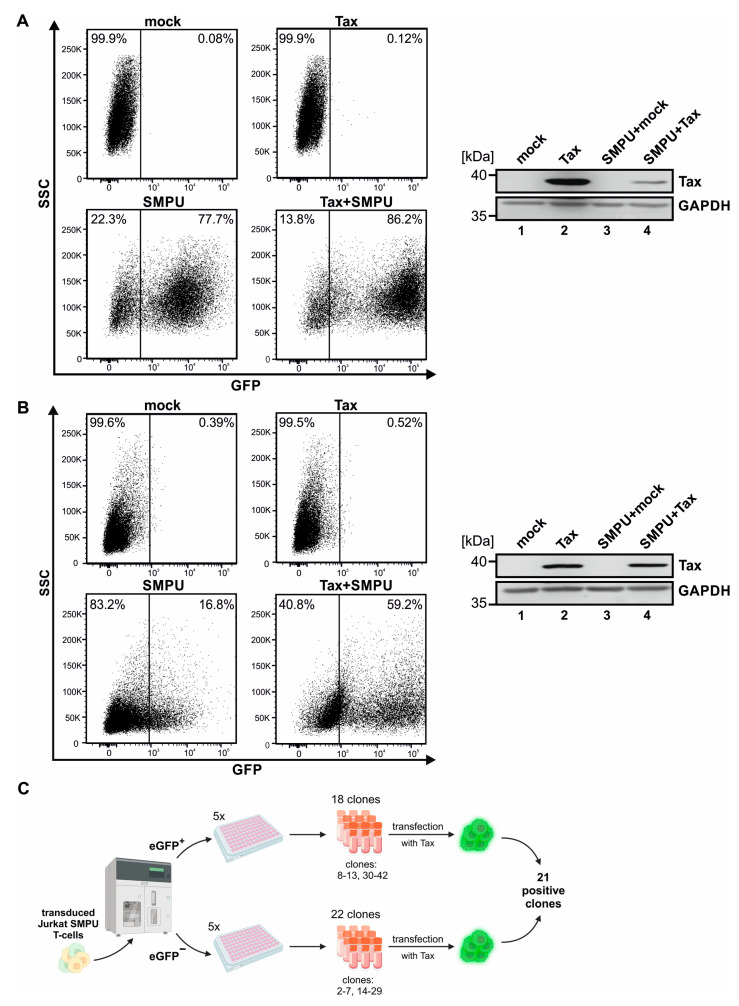
Generation of a Tax-responsive Jurkat–SMPU reporter cell line. (**A**,**B**) Transient expression of the SMPU-18 × 21-eGFP reporter. First, (**A**) 293T or (**B**) Jurkat T-cells were transiently transfected with either the pSG5 empty vector, the Tax expression vector pSG5-Tax, the SMPU-18 × 21-eGFP reporter vector, or co-transfected with pSG5-Tax and SMPU-18 × 21-eGFP. Flow cytometry (**left** side) and Western Blot analysis (**right** side) were performed at 48 h post-transfection. For flow cytometry, forward and side scatter (SSC) were used to gate on living cells. The resulting gates are illustrated, and GFP is plotted against SSC. Western Blots of Tax and GAPDH are depicted. (**C**) Schematic overview on cell sorting of Jurkat T-cells after transduction. Sorting resulted in 40 individual clones (22 were initially eGFP^−^, 18 had background eGFP signal (eGFP^+^) during sorting). Clones were tested for their Tax responsiveness by transient transfection with pSG5-Tax, which led to the identification of 21 Tax-responsive clones. Created with BioRender.com.

To generate a Tax-responsive reporter cell line, we first confirmed the Tax responsiveness of the SMPU-18 × 21-eGFP reporter (SMPU) in two different cell lines. For this purpose, 293T cells were transiently transfected with either the pSG5 empty vector, the Tax expression vector pSG5-Tax, SMPU, or co-transfected with pSG5-Tax and SMPU. Upon transient transfection of 293T cells with SMPU, already 77% of cells expressed eGFP (Figure 1A, lower left dot plot) compared to mock-transfected cells (Figure 1A, upper left dot plot). Co-expression of Tax increased not only the frequency of eGFP-expressing cells but also their mean fluorescence intensity, resulting in 86% of eGFP-positive cells (Figure 1A, lower right dot plot). Western Blot analysis confirmed robust expression of Tax (Figure 1A, right). Since the background signal of eGFP expression was very high in 293T cells even in the absence of Tax, we next tested Jurkat T-cells, a cell line that is more relevant to HTLV-1 infection in vivo.

Comparable to 293T cells, Jurkat T-cells were transiently transfected with either the pSG5 empty vector, the Tax expression vector pSG5-Tax, SMPU, or co-transfected with pSG5-Tax and SMPU. Transient transfection of Jurkat T-cells with SMPU resulted in 17% of eGFP background expression (Figure 1B, lower left dot plot) compared to mock-transfected cells (Figure 1B, upper left dot plot). Co-expression of Tax, confirmed by Western Blot (Figure 1B, right), increased both the frequency of eGFP-expressing cells as well as their mean fluorescence intensity, resulting in 59% of eGFP-positive cells (Figure 1B, lower right dot plot). Although the observed background activity of the SMPU reporter was lower in Jurkat than in 293T cells upon transient transfection (compare Figure 1B to Figure 1A, lower left dot plots), it would be challenging to distinguish the background signal from true positive results upon infection of cells with HTLV-1 since HTLV-1 infection of Jurkat T-cells occurs at low frequencies [28,37]. To reduce the background activity of the SMPU reporter vector and to eliminate the necessity of transient transfection steps, Jurkat T-cells were stably transduced with the SMPU reporter vector. After confirmation of a lowered background activity of the stably transduced SMPU reporter upon transient transfection with pSG5 empty vector (3%) and a potent Tax responsiveness upon transfection with pSG5-Tax (21%), the polyclonal Jurkat–SMPU T-cell line was subjected to cell sorting to select for single cells with low background activity (Figure 1C). Subsequently, 40 individual clones were expanded, with 22 of them exhibiting no eGFP signal initially, while 18 clones presented a weak background eGFP signal. All 40 generated Jurkat–SMPU T-cell clones were further tested for their Tax responsiveness via transient transfection with either pSG5-Tax, with the CREB-deficient Tax point mutant pSG5-M47, which is impaired in transactivation [34], or the pSG5 empty vector. In summary, 21 of the single-cell clones displayed eGFP-positive cells (ranging from 6.3 to 60.5% of cells), thus reflecting an appropriate response to transient transfection with Tax (Figure S1). From the 21 Tax-responding clones, we selected clones 21, 29, 31, and 34 for further evaluation. These clones had no background eGFP expression upon transient transfection with the pSG5 empty vector, displayed only moderate background reporter activity upon transfection with the CREB-deficient M47 mutant, and exhibited some of the highest eGFP expression levels upon transfection with pSG5-Tax of all tested clones (Figure S1).

### 3.2. Characterization of Tax Responsiveness in Reporter T-Cell Clones

**Figure 2:**
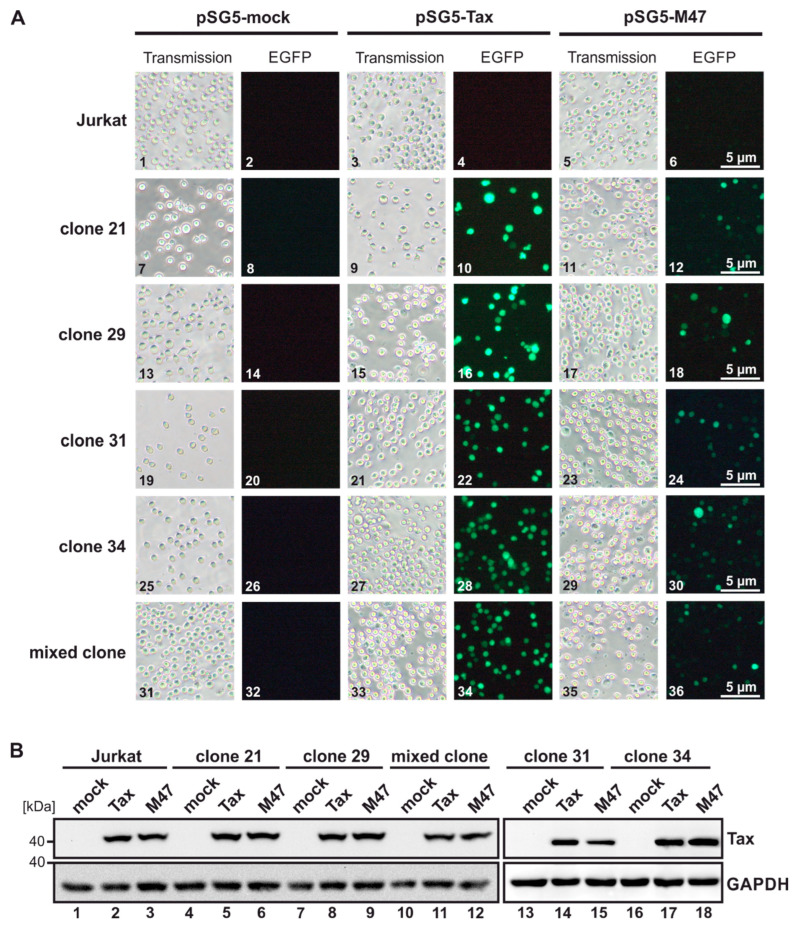
Tax activates GFP expression in Jurkat–SMPU reporter T-cell clones at higher levels than the CREB-deficient Tax mutant M47 despite comparable expression levels. Jurkat T-cells, Jurkat–SMPU clones 21, 29, 31, and 34, and a mixed clone of the respective clones were transfected with either the wildtype expression plasmid pSG5-Tax, with the expression plasmid of the CREB-deficient Tax mutant M47 (pSG5-M47), or with the pSG5 empty vector. (**A**) eGFP expression was analyzed at 48 h post-transfection using a Nikon Eclipse TS2-FL inverted fluorescence microscope and TCapture software. (**B**) Representative Western Blots at 48 h post-transfection depicting Tax and GAPDH.

To further demonstrate Tax responsiveness and the ability efficiency to activate eGFP expression after the initial pre-screening, the selected Jurkat–SMPU reporter T-cell clones 21, 29, 31, 34, and a mixed clone of the respective clones were transiently transfected with the pSG5 empty vector, pSG5-Tax, or with the CREB-deficient Tax mutant pSG5-M47. Epifluorescence imaging 48 h post transient transfection revealed eGFP expression in all Tax-transfected SMPU single-cell clones and the mixed clone (Figure 2A, 10, 16, 22, 28, 34) and to a lower extent in M47-transfected clones (Figure 2A, 12, 18, 24, 30, 36), while mock-transfected SMPU clones remained negative for eGFP expression (Figure 2A, 8, 14, 20, 26, 32), precluding unspecific eGFP activation. The parental wildtype Jurkat T-cell line remained eGFP-negative as expected, independent of the expression plasmids expressed (Figure 2A, 2, 4, 6). Thus, the selected Jurkat–SMPU reporter clones are Tax-inducible and eGFP is expressed after transient transfection in the selected clonal or mixed cell lines. Western Blot analysis confirmed Tax expression at comparable levels in cells transfected with pSG5-Tax or pSG5-M47 (Figure 2B). Although fluorescence imaging showed no differences in eGFP expression between the four tested single-cell clones (Figure 2A), clones 31 and 34 displayed high background eGFP levels in co-culture experiments, thus limiting further experiments to clones 21 and 29.

**Figure 3:**
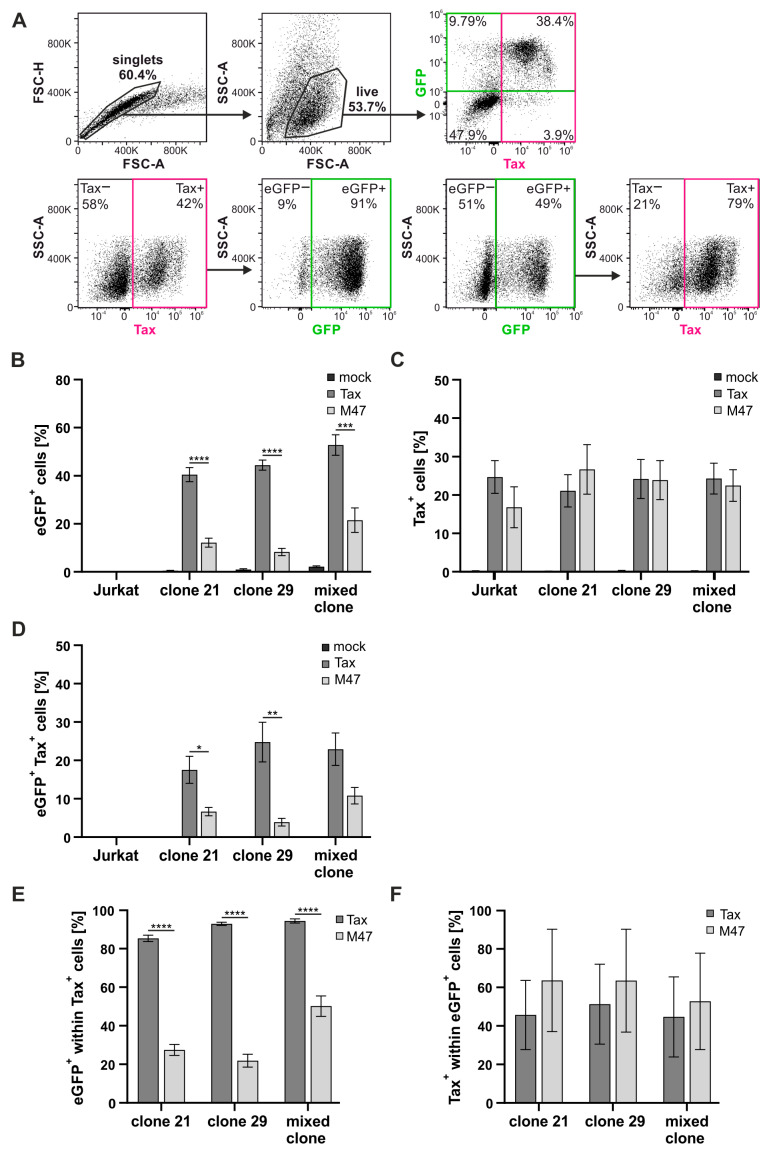
Jurkat–SMPU reporter T-cell clones are Tax-responsive and show high efficiency in activating eGFP expression. Jurkat T-cells, Jurkat–SMPU reporter T-cell clones 21, 29, and the mixed clone of 21, 29, 31, and 34 were transfected with either the wildtype expression plasmid pSG5-Tax, with the expression plasmid of the CREB-deficient Tax mutant M47 (pSG5-M47), or with the pSG5 empty vector. After 48 h, cells were fixed, intracellularly stained with primary anti-Tax and secondary anti-mouse IgG AF647-coupled antibodies, and analyzed via flow cytometry. (**A**) Gating strategy depicting representative dot plots of clone 29 transfected with pSG5-Tax. Upper row: Cells were plotted as size (FSC-A; forward scatter area) against size (FSC-H; forward scatter height) to exclude doublets. Then, living cells were determined by plotting size (FSC-A) against granularity (SSC-A; side scatter area). Within the live cell gate, eGFP- and Tax-positive cells were assessed. Lower row, left: After gating for Tax-positive cells by plotting Tax against granularity (SSC), the eGFP-positive cells within the Tax-positive population were assessed. Lower row, right: After gating for eGFP-positive cells by plotting GFP against SSC, the Tax-positive cells within the eGFP-positive population were assessed. (**B**) eGFP-positive, (**C**) Tax-positive, and (**D**) both eGFP- and Tax-positive cells, as well as (**E**) eGFP-positive cells within the Tax-positive population and (**F**) vice versa were assessed by flow cytometry analysis. Mean values of five independent experiments ± SE are depicted. Data were analyzed for normal distribution using the Shapiro–Wilk Test followed by (**B**,**D**) one-way ANOVA and Tukey’s test (more than two groups) or (**E**) an unpaired Student’s *t*-test (two groups) (* *p* ≤ 0.05, ** *p* ≤ 0.01, *** *p* ≤ 0.001, **** *p* ≤ 0.0001).

To quantitatively evaluate eGFP expression in parallel to Tax expression within the same cell, the activity of the reporter and the intracellular expression of Tax were analyzed in parallel by flow cytometry. As depicted in the representative dot plot (Figure 3A), doublets were excluded, and living cells were determined within the singlets gate. In the living cell gate, cells positive for both eGFP and Tax were assessed by plotting Tax against GFP (Figure 3A, upper row). Further, Tax or GFP was plotted against granularity (side scatter; Figure 3A, bottom row). To quantify the efficiency of inducing eGFP expression in Tax-positive cells, eGFP-positive cells within the Tax-positive population were assessed (Figure 3A, bottom row, left side). In addition, the frequencies of Tax-positive cells within the eGFP-positive population were quantitated (Figure 3A, bottom row, right side).

Transfection of cells with the empty vector pSG5 resulted in a low background eGFP expression in the mixed clone only while background eGFP expression was barely detectable in the Jurkat–SMPU reporter T-cell clones 21 and 29 (Figure 3B), thus confirming the data of the pre-screening (Figure S1). In contrast, transfection with pSG5-Tax resulted in approximately 40% of eGFP-positive cells in clones 21 and 29 and over 50% of eGFP-positive cells in the mixed clone. Although transfection with pSG5-M47 resulted in higher eGFP expression in the mixed clone compared to the reporter clones 21 and 29, eGFP expression was significantly lower after transfection with pSG5-M47 compared to pSG5-Tax in all clones tested (*p* < 0.001; Figure 3B). In the parental Jurkat T-cell line, there was no eGFP expression detectable in all conditions. All cells transfected with pSG5-Tax or pSG5-M47 showed transfection efficiency of approximately 20% Tax-positive cells, confirming that comparable amounts of Tax or M47 expression in the cells result in different amounts of eGFP expression (Figure 3C). All tested Jurkat–SMPU reporter T-cell clones (clone 21, 29, and mixed clone) showed a significantly higher frequency of eGFP and Tax double-positive cells after transfection with pSG5-Tax compared to the transfection with pSG5-M47 (Figure 3D). The eGFP and Tax double-positive cell population was expectably absent when cells were transfected with the empty vector pSG5 or when the parental Jurkat T-cells were transfected.

In all tested Jurkat–SMPU reporter clones, the portion of eGFP-positive cells within the Tax-positive population was high—ranging from 83.5 to 92.9%—and thereby significantly higher compared to clones transfected with pSG5-M47 (Figure 3E). These results indicate efficient reporter activity of the Jurkat–SMPU reporter vector in the tested single-cell clones upon expression of Tax. However, it is noteworthy that the mixed clone transfected with pSG5-M47 had a higher proportion of eGFP-positive cells within the Tax-positive population compared to the reporter clones 21 and 29. Interestingly, the frequency of Tax-positive cells within the eGFP-positive populations was only between 40 and 60% and was, despite high variations, comparable between the reporter cell clones transfected with pSG5-Tax or pSG5-Tax-M47 (Figure 3F). Thus, we observed the activity of eGFP, while Tax protein was undetectable by intracellular flow cytometry, which may be explained by different sensitivity of the detection method, prolonged half-life of the eGFP reporter signal compared to Tax protein expression, or induction of the reporter independent of Tax protein. Summed up, the Jurkat–SMPU reporter clones 21 and 29 as well as the mixed clone exhibit high efficiency in activating GFP in the presence of Tax protein.

### 3.3. Mitogenic Stimulation Identifies Reporter T-Cell Clones with Low Background Activity

**Figure 4:**
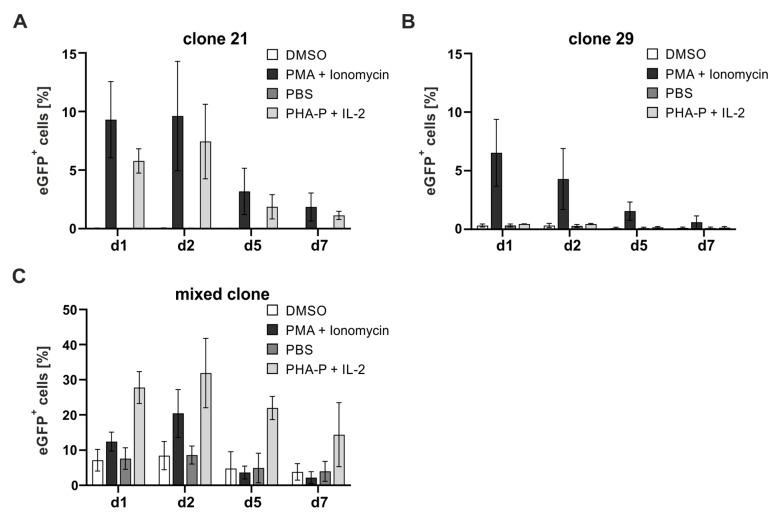
Determination of background reporter activity after stimulation of Jurkat–SMPU reporter T-cell clones using different stimuli. (**A**) Jurkat–SMPU reporter clone 21, (**B**) clone 29, or (**C**) the mixed clone of clones 21, 29, 31 and 34 were stimulated with PMA (20 nM) and Ionomycin (1 µM), PHA-P (5 µg/mL) and IL-2 (25 U/mL) or respective solvent controls (DMSO, PBS). Cells were analyzed after d1, d2, d5, and d7 of stimulation. GFP-positive cells were assessed by FACS analysis. Mean values of three independent experiments ± SD are depicted.

The ultimate goal of this study was to use the novel reporter cell clones as a readout system for HTLV-1 infection, which requires several days of co-culture with chronically infected T-cells [28,37]. However, infection is more efficient in activated acceptor T-cells due to the upregulation of HTLV-1 receptor components following T-cell stimulation [10,11]. To exclude unspecific activation of the *U3R*-driven eGFP reporter upon mitogenic stimulation of Jurkat T-cells and to select appropriate mitogens for infection experiments, the Jurkat–SMPU reporter clones 21 and 29 and the mixed clone of the clones 21, 29, 31, and 34 were either treated with the mitogen PMA (20 nM) in combination with the Ca^2+^ ionophore ionomycin (1 µM) or with the lectin PHA-P (2 µg/mL) together with IL-2 (25 U/mL) and compared to the respective solvent controls DMSO or PBS at one, two, five, or seven days post-treatment (Figure 4). Flow cytometry revealed that mitogenic stimulation of clone 21 induced eGFP expression ranging from 5.8% (PHA-P/IL-2) to 9.6% (PMA/ionomycin) of cells on days 1 and 2 post-stimulation (Figure 4A), and signals declined to values between 1.1% (PHA-P/IL-2) and 3.2% (PMA/ionomycin) of eGFP-positive cells on days 5 and 7. In contrast, treatment with PHA-P and IL-2 induced hardly any eGFP expression in clone 29, while PMA and ionomycin led to eGFP expression in 6.5% of cells after one day, which gradually declined again over 7 days to 0.61% of eGFP-positive cells (Figure 4B). Thus, activation with PHA-P and IL-2 induces lower background activity of the reporter in clone 21 and no background signals in clone 29 compared to PMA/ionomycin treatment, suggesting the usage of PHA-P and IL-2 to stimulate the cells in further experimental settings. Stimulation of the mixed clone led to eGFP expression independent of the mitogens used ranging up to 31.9% of eGFP-positive cells (Figure 4C). Moreover, the mixed clone already exhibited high background activity when cultured in the presence of the solvent controls, which first increased from day one to day two and then decreased again until day 7, but it never fell below 3.8% eGFP-positive cells (day 7). Therefore, the mixed clone of clones 21, 29, 31, and 34 is not suitable as a tool to monitor HTLV-1 infection due to its high background signals. These results also underline the importance of (1) selecting an appropriate mitogen and (2) generating single-cell clones after transduction of the reporter. Together, Jurkat–SMPU reporter cell clones 21 and 29 stimulated with PHA-P and IL-2 were identified as a suitable cell culture system to be used in infection experiments due to their Tax-responsiveness (Figure 2A, Figure S1 and Figure 3B,D,E), high efficiency in activating GFP expression (Figure 3A,B,E), and limited background activity (Figure 4A,B).

### 3.4. Reporter Cell Clones Are Suitable to Monitor HTLV-1 Infection

**Figure 5:**
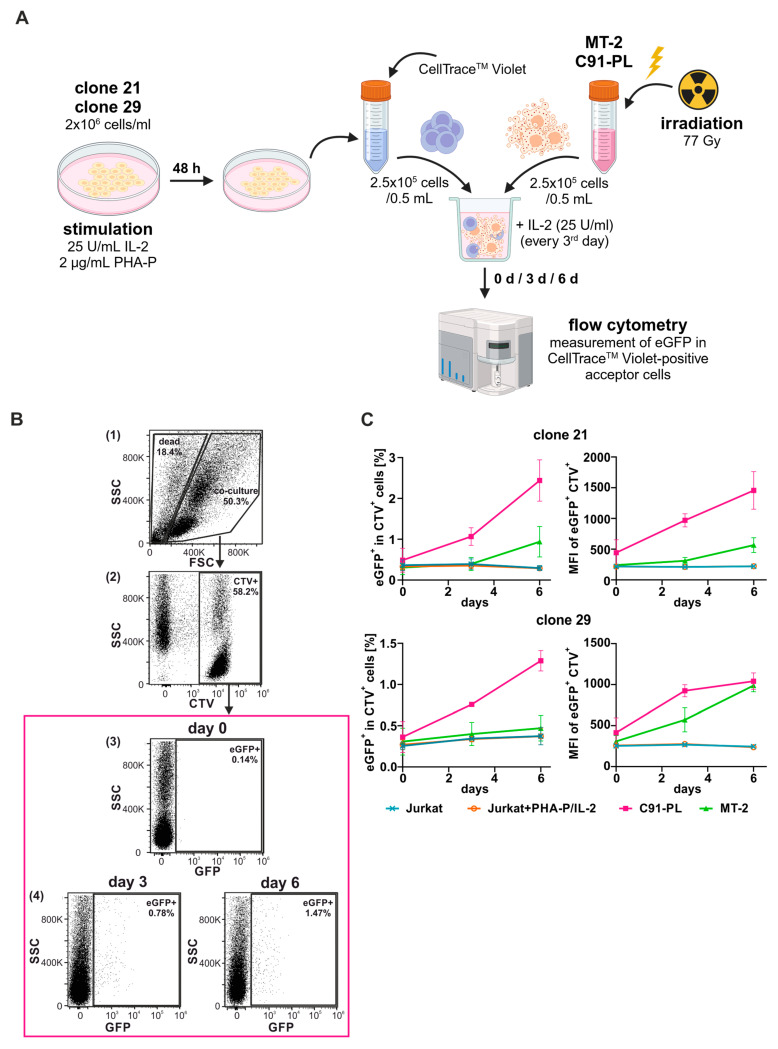
Jurkat–SMPU reporter T-cell cones 21 and 29 are suitable tools to study infection using C91-PL and MT-2 cells as donor cells. (**A**) Schematic overview of experimental setup. Jurkat–SMPU reporter T-cell clones 21 and 29 were stimulated with 25 U/mL IL-2 and 2 µg/mL PHA-P. After 48 h, stimulated cells were stained with CellTrace^TM^ Violet (CTV) and used as acceptor cells in co-culture experiments. Before co-culture, donor cells were irradiated (77 Gray (Gy)). On days 0 and 3 of co-culture, cells were additionally stimulated with 25 U/mL IL-2. Flow cytometry analysis of eGFP reporter activity in CTV^+^ cells was performed on days 0, 3, and 6 of co-culture. As negative controls, donor cells were replaced with either stimulated or unstimulated Jurkat T-cells (not depicted). Created with BioRender.com. (**B**) Gating strategy illustrating representative dot plots of clone 29 co-cultured with irradiated C91-PL donor cells for 3–6 days. (1) Cells were first plotted size (FSC; forward scatter area) against granularity (SSC; side scatter) to determine living cells of co-culture. (2) Within the co-culture gate, CTV-positive cells were assessed. (3,4) Infection of CTV^+^ Jurkat–SMPU clones was determined by plotting the CTV^+^ gate against granularity (SSC) on days 0, 3, and 6 of co-culture (highlighted in pink box). (**C**) Flow cytometry analysis depicting the frequency of eGFP-positive cells within the CTV^+^ population (**left** column) or mean fluorescent intensity (MFI) of eGFP^+^ CTV^+^ cells (**right** column). Mean values of three independent experiments ± SE are depicted.

Applying cell culture conditions from Figure 4, the Jurkat–SMPU reporter T-cell clones 21 and 29 were utilized in co-culture experiments to ultimately define their suitability to study HTLV-1 infection (Figure 5A). Therefore, the acceptor SMPU cells were stimulated with PHA-P and IL-2 two days prior to co-cultivation with irradiated (77Gy) chronically HTLV-1-infected donor T-cells, and additionally, IL-2 was supplemented every two days. Immediately prior to co-culture, acceptor SMPU reporter T-cells were stained with CTV, a succinimidyl ester dye being not transferred to bystander cells in culture and allowing monitoring of acceptor cells over several generations [38,39]. At different time points, GFP^+^ CTV^+^ acceptor reporter cells within the co-cultures were analyzed by flow cytometry analysis. Figure 5B depicts a representative gating strategy with (1) exclusion of dead cells, (2) gating for CTV-specific fluorescence, and (3–4) evaluation of GFP-positive cells within the CTV-positive population on day zero, three, and six of co-culture, indicating HTLV-1 infection. The frequency of eGFP-positive cells within the CTV-positive population (Figure 5C, left column) or mean fluorescent intensity (MFI) of eGFP^+^ CTV^+^ cells (Figure 5C, right column) were analyzed.

In both clones 21 and 29, basal eGFP expression levels within CTV-positive cells upon co-culture with HTLV-1-negative cells donor cells (stimulated or unstimulated Jurkat T-cells) were relatively low and did not exceed 0.4% (0.25 to 0.39%) or an MFI of 280 arbitrary units (AU) (210 to 276 AU). Upon co-culture with the chronically HTLV-1-infected cell line C91-PL, Jurkat–SMPU reporter cell clone 21 already exhibited 1.1% of eGFP-positive cells within the CTV-positive population on day three of co-culture, which was also represented by an increase in the MFI (from 442 AU on day 0 to 969 AU on day 3). After six days of co-culture, up to 2.4% of reporter cells were eGFP-positive, thus reflecting infected cells, and the MFI of eGFP increased to its maximum of 1455 AU. When using reporter cell clone 29 as acceptor cells, the frequency of eGFP-positive cells only marginally increased after three days of co-culture with C91-PL cells to 0.76%, but it further increased on day six, reaching frequencies of 1.29% eGFP-positive cells. Compared to clone 21, the MFI of clone 29 also first increased from day zero to day three, but other than the MFI of clone 21, it did not change a lot between day three (924 AU) and day six (1040 AU), suggesting that more cells are infected within clone 29, but with less virus.

In line with previous reports using different reporter vectors [28], the frequencies of eGFP-positive cells within the CTV-positive population in both clones 21 and 29 were lower when MT-2 cells were used as HTLV-1-infected donor cells compared to C91-PL cells (Figure 5C, compare green (MT-2) with pink (C91-PL)). On day three of co-culture, both clones 21 and 29 showed only background fluorescence, but on day six, clone 21 exhibited 0.9% eGFP-positive cells, while in clone 29, the frequency of eGFP-positive cells was close to the background (Figure 5C, green). Clone 21 showed a quite similar development when analyzing the MFI over the time course, but clone 29 already showed a slightly increased MFI of 571 AU on day three, which further increased on day six to the same level observed after co-culture with C91-PL cells to 988 AU.

In summary, both Jurkat–SMPU reporter cell clones 21 and 29 are suitable tools to robustly report infection by both HTLV-1-infected C91-PL and MT-2 donor cells on the single-cell level; however, clone 21 and C91-PL cells result in higher eGFP expression levels and might therefore be favored in infection experiments.

## 4. Discussion

HTLV-1 transmits via close cell–cell contacts, and its transmission levels are inefficient compared to other retroviruses like the related lentivirus human immunodeficiency virus type-1 (HIV-1), which is also able to infect cells through cell-free particles [2,40]. To measure HTLV-1 cell-to-cell transmission, several *LTR*-based reporter vectors have been described including *LTR*-driven reporter cells [28], *U3R*-driven luciferase reporter cells [23], viral *CRE*-driven luciferase plasmids [29], or *LTR*-driven NanoLuc reporter systems [30]. Upon infection with HTLV-1 and subsequent generation of the viral transactivator protein Tax, reporter cell activity increases, thus serving as a readout for viral replication and HTLV-1 infection. Although being useful in delineating the molecular details of HTLV-1 cell-to-cell transmission, previously used reporter systems face some limitations, which we sought to overcome in this study. Hence, we used a transduced and chromosomally integrated reporter system derived from a self-inactivating lentivirus vector developed earlier [31]. Briefly, the 18 × 21 bp-eGFP SMPU reporter vector carries an expression cassette for enhanced green fluorescent protein (eGFP) under the control of an 18-fold amplified 21 bp Tax-responsive element (TRE) and has been used previously to monitor Tax-induced transactivation [31,41]. We generated a Jurkat 18 × 21 bp reporter T-cell line (Jurkat–SMPU) by lentiviral transduction. Single-cell clones were selected by FACS sorting, and out of 40 clones, the two most promising ones (#21 and #29) were further characterized in this study. Thus, our novel reporter cells Jurkat–SMPU (1) allow discrimination of single cells by monitoring of eGFP by flow cytometry or immunofluorescence, (2) use an artificial but amplified viral promoter stretch (18 × 21 bp), which may also monitor rare events of virus transmission, and (3) are derived from single-cell clones, thus allowing easier reproducibility of data compared to polyclonal cell lines.

To further characterize the reporter cells, we first analyzed the Tax responsiveness of Jurkat–SMPU and compared a mixed clone with single-cell clones. Upon expression of Tax or the CREB-deficient Tax mutant M47, GFP signals were monitored via fluorescence microscopy and flow cytometry, confirming that Tax indeed activates the 18 × 21 bp promotor while M47 is impaired as shown previously [34]. This is in line with CREB’s role in building a protein complex with Tax and CBP, which is essential for the activation of the viral promotor [12]. Compared to other studies, we not only quantitated the frequency of reporter-positive cells but also assessed the frequency of Tax-positive cells by intracellular staining of Tax protein followed by flow cytometry to analyze Tax responsiveness and efficiency to induce TRE-driven eGFP expression. Clones transfected with Tax revealed over 80% GFP-positive cells within the Tax-positive population, confirming a high efficiency of Tax to activate the reporter, while upon transfection of the CREB-deficient Tax mutant M47, the frequency of GFP-positive cells in the Tax-positive population decreased significantly. Moreover, confirmatory experiments using Western Blot and FACS analysis revealed that the differences in reporter activity following Tax and M47 expression cannot be attributed to differences in protein expression levels, which were comparable. Since Tax is a potent inducer of HTLV-1 transcription, we expected most cells within the GFP-positive populations to be Tax-positive. However, against expectation, only approximately half of the cells within the GFP-positive population were Tax-positive after transfection with Tax or M47, as detected by intracellular flow cytometry. This may be explained by (1) different sensitivity of the detection methods, (2) prolonged half-life of the eGFP reporter signal compared to Tax protein expression, (3) prolonged transcription of retrovirally stably transduced eGFP expression cassettes compared to transiently transfected Tax expression vectors, and (4) induction of the reporter independent of Tax protein. In more detail, (1) Tax shuttles between the cytoplasm and the nucleus and translocates into the nucleus very quickly after synthesis [42,43]. Since the nucleus is more challenging to access, even with permeabilization techniques, Tax protein might be less detectable by intracellular staining compared to the detection of a self-fluorescent eGFP protein. (2) Tax might get degraded more rapidly than eGFP, resulting in cells that still express eGFP but no longer Tax. This is in line with studies measuring the half-lives of GFP (T1/2 = 26 h) [44] and Tax, whose metabolic half-life varies between 15 and 24 h, depending on the methods and cell types used [35,42]. (3) While Tax was transiently transfected, and its transcription is driven by an SV40-driven promoter, *eGFP* transcription is driven by an integrated vector and thought to initiate after Tax has been expressed and might thus be more durable. (4) Finally, it might be possible that not just Tax protein is able to induce the expression of the reporter gene but also *Tax* RNA. Functionally active RNA has been described for the related HTLV-1-encoded protein HBZ, which promotes T-cell proliferation in its RNA conformation [45].

T-cell activation is a pre-requisite for efficient and productive HTLV-1 infection in vitro since the HTLV-1 receptor components Glut-1 and Nrp-1 are upregulated following T-cell stimulation [10,11]. However, T-cell activation might also impact the background activity of *LTR*- or *U3R*-driven reporter cells used as readout for HTLV-1 cell-to-cell transmission due to activating CREB-dependent signaling. Since HTLV-1 infection is rather inefficient when compared to other retroviruses [40], the reporter cells should not respond to the mitogens supplied during cell-to-cell infection assays. Thus, we compared the background levels of eGFP expression in the newly generated SMPU reporter cells upon mitogenic stimulation with PMA/ionomycin and the lectin PHA-P in combination with the T-cell growth factor IL-2. Ionomycin synergizes with PMA to activate NF-κB via protein kinase C, and it activates Ca^2+^/calmodulin-dependent signaling [46]. Our study revealed that the mixed reporter cell clone was highly responsive towards mitogenic activation independent of the mitogen used, which may be due to a mixture of different integration sites of the reporter gene cassette. Moreover, mitogenic stimulation of single-cell clones with PMA/ionomycin induced a higher background activity of the SMPU reporter, probably due to the fact that the activation of CREB-dependent signaling pathways also took place due to the application of the Ca^2+^ ionophore ionomycin [47]. Contrarily, both single-cell Jurkat–SMPU clones exhibited much less (#21) to non-existent (#29) background eGFP activity following activation with PHA-P/IL-2, thus identifying suitable candidate clones and appropriate mitogens for infection experiments.

HTLV-1 transmission and infection of T-cells require cell–cell contacts. However, the quantification of reporter gene activity does not exclusively display infection as the transactivation of the reporter can also occur through cell fusion and syncytia formation or by delivery of Tax via extracellular vesicles [2,48]. To exclude passive Tax protein transfer on the one hand and the outgrowth of productively-infected donor cells on the other hand, HTLV-1-infected donor cells were gamma-irradiated before co-culture with the Tax-responsive Jurkat–SMPU reporter cells, similar to previous reports [28,49]. Additionally, we also labeled the Jurkat–SMPU reporter cells with a succinimidyl ester live cell dye, which is not transferred to bystander cells in culture and allows for monitoring of the reporter cells over several generations [38,39]. The latter is advantageous since it allows for the monitoring of HTLV-1 infection on a single-cell level and opens the door for follow-up studies with sorted acceptor cells.

When measuring HTLV-1 infection in vitro, we detected robust but low levels of reporter gene activity after six days of co-culture, which is in line with previous reports using different reporter systems [28,50]. Compared to previous studies using single-cell clones after the selection of LTR-GFP plasmid-transfected cells [28], we always included co-cultures with uninfected T-cells to exclude unspecific activation of the integrated reporter either by the mitogens or the co-cultured donor cells supplemented. Interestingly, previous studies also detected higher frequencies of GFP-positive acceptor cells and different kinetics of reporter gene activity [28]. While we used retrovirally transduced and integrated reporter vectors, it cannot be concluded whether the reporter vectors from previous studies were maintained as extrachromosomal elements or integrated into the genome as well [28]. In line with earlier work [4,28], we found that chronically infected C91-PL cells induce higher levels of reporter gene activity compared to MT-2 cells, suggesting that C91-PL transmits more virus. This may be explained by the fact that C91-PL is known to transmit predominantly via viral biofilms, while MT-2 is supposed to harbor less viral biofilm, but is able to induce tunneling nanotubes for viral transmission [4,6,24,28].

Next to reporter cell lines as reported here and elsewhere [23,28,29,30], also other systems like single-cycle, replication-dependent reporter systems have been developed to monitor HTLV-1 cell-to-cell transmission [40,50]. This method is based on virus-like particles (VLPs) containing reporter genes that allow for the quantitative measurement of cell-to-cell infection. While being an elegant system to measure reporter activity upon de novo infection and reverse transcription only, the single-cycle, replication-dependent reporter systems also have some limitations: (1) they require transfection of the respective plasmids for every single experiment, while when using a clonal reporter cell line, only cell expansion is required; (2) the transcriptional regulation of the packaging plasmids used to produce viral particles differs from that of authentic HTLV-1 since the 5′*LTR* and the 5′ untranslated region are replaced by a CMV promoter, which is fused to a fragment of the R region of the *LTR* [51], while chronically infected cell lines, as used in our study, use the authentic HTLV-1 promoter; and (3) in several studies, pseudotyped reporter vectors with VSV-G were used instead of using authentic Env, thus changing the tropism of HTLV-1 viruses and limiting the interpretation of obtained data [52].

To sum up, the Jurkat–SMPU reporter T-cell line is a novel suitable tool to monitor HTLV-1 infection on the single-cell level, albeit confirming that HTLV-1 infection is very low.

## Data Availability

The original contributions presented in this study are included in this article/Supplementary Material. Further inquiries can be directed to the corresponding author.

## Supplementary Materials

The following supporting information can be downloaded at https://www.mdpi.com/article/10.3390/pathogens13111015/s1: Figure S1: Tax activates GFP expression in Jurkat-SMPU reporter T-cell clones at higher levels than the CREB-deficient Tax mutant M47.
