# Supplementary material for: A Novel Tax-Responsive Reporter T-Cell Line to Analyze Infection of HTLV-1
Source: Pathogens. 2024 Nov 19;13(11):1015. doi: 10.3390/pathogens13111015 (PMC11597676; doi:10.3390/pathogens13111015)
Supplement: Supplementary file 1 [file pathogens-13-01015-s001.zip › pathogens-3262212-supplementary.pdf]

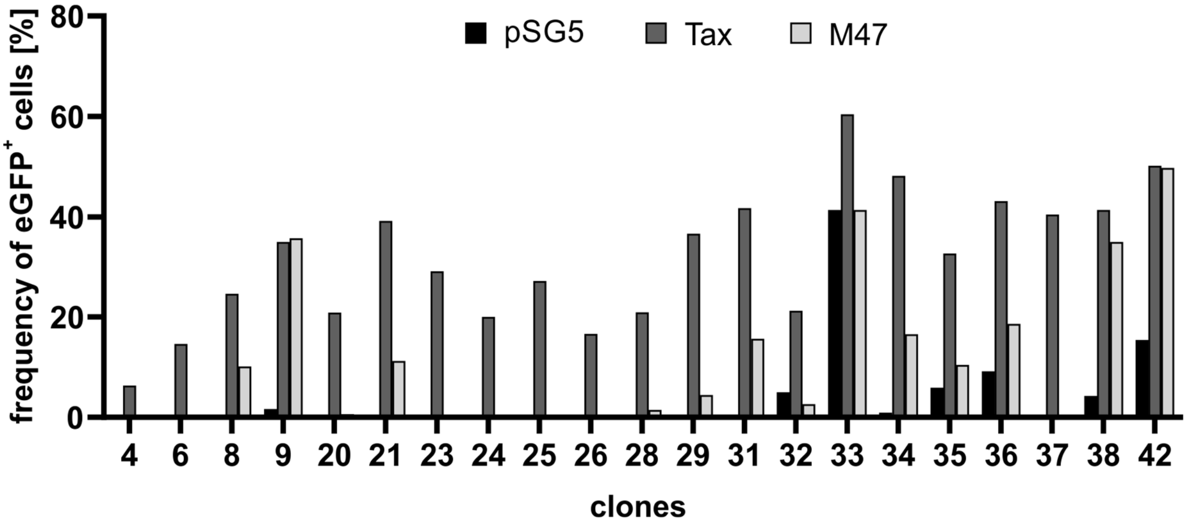

Figure S1. Tax activates GFP expression in Jurkat-SMPU reporter T-cell clones at higher levels than the CREB-deficient Tax mutant M47. Sorted Jurkat-SMPU clones as indicated were transfected with either the wildtype expression plasmid pSG5-Tax, with the expression plasmid of the CREB deficient Tax mutant M47 (pSG5-M47) or with the pSG5 empty vector. eGFP expression was analyzed at 48h post transfection using flow cytometry. 40 individual clones were tested, 21 clones displaying eGFP positive cells after transfection of pSG5-Tax (ranging from 6.3 to 60.5% of cells) are shown
